# Use of a choice survey to identify adult, adolescent and parent preferences for vaccination in the United States

**DOI:** 10.1186/s41687-019-0135-0

**Published:** 2019-07-29

**Authors:** Tara A. Lavelle, Mark Messonnier, Shannon Stokley, David Kim, Aparna Ramakrishnan, Achamyeleh Gebremariam, Norma-Jean E. Simon, Angela M. Rose, Lisa A. Prosser

**Affiliations:** 1 Center for the Evaluation of Value and Risk in Health (CEVR), Institute for Clinical Research and Health Policy Studies, Tufts Medical Center, 800 Washington St, Boston, MA 02111 USA; 2 National Center for Immunization and Respiratory Diseases, Centers for Disease Control and Prevention, 1600 Clifton Road, Atlanta, GA 30329 USA; 3 Devi Partners, LLC, 112 Madera Ave, San Carlos, CA 94070 USA; 4 Susan B. Meister Child Health Evaluation and Research Center, Department of Pediatrics, Medical School, University of Michigan, 300 North Ingalls St, Ann Arbor, MI 48109 USA; 5 Ann and Robert H. Lurie Children’s Hospital, 225 East Chicago Ave, Chicago, IL 60611 USA; 6 Department of Health Management and Policy, School of Public Health, University of Michigan, 1415 Washington Heights, Ann Arbor, MI 48109 USA

**Keywords:** Vaccination, immunization rates, Stated preferences, Adolescent, Parents, Adults

## Abstract

**Background:**

Adult and adolescent vaccination rates are far below coverage targets in the United States. Our objective was to identify the most influential factors related to vaccine uptake among adults, adolescents, and parents of adolescents (parents) in the United States.

**Methods:**

We used a fractional factorial design to create a binary choice survey to evaluate preferences for vaccination. The national survey was fielded to a sample of adults, adolescents ages 13–17 years, and parents, using a national probability-based online research panel in November 2015. Respondents were presented with 5 profiles of a hypothetical vaccine and asked in a series of questions whether they would accept each vaccine. We analyzed the binary choice data using logistic regression in STATA v13 (College Station, TX) to calculate the odds that a participant would choose to accept the vaccine.

**Results:**

We received completed responses from 334 (51%) of 652 adults, 316 (21%) of 1516 adolescents, and 339 (33%) of 1030 parents. Respondents were generally representative of the U.S. population. Vaccine effectiveness was the most influential factor in the choice to vaccinate for all groups. Other most influential factors were primary care provider (PCP) recommendation and the out-of-pocket cost of the vaccine. Other factors such as risk of illness, risk of vaccine side effects, vaccination location, and time for vaccination were not important in the decision to get vaccinated.

**Conclusions:**

Adults, adolescents, and parents are most sensitive to vaccine effectiveness, PCP recommendation, and out-of-pocket cost for vaccination in their decision to get vaccinated. Strong PCP recommendations that focus on vaccine effectiveness and health care policies that minimize out-of-pocket costs for vaccinations may increase vaccine uptake by adults and adolescents.

**Electronic supplementary material:**

The online version of this article (10.1186/s41687-019-0135-0) contains supplementary material, which is available to authorized users.

## Introduction

Achieving target coverage rates for many adolescent and adult vaccines remains a public health challenge in the United States. Vaccination rates for influenza, human papillomavirus (HPV), varicella, and hepatitis B have fallen short of national immunization goals in recent years. [1] [2, 3]. Previous research has identified numerous factors influencing vaccine uptake, including knowledge of the vaccine and illness [4, 5], physician recommendation [5–8], perception of the vaccine’s effectiveness [6, 9], risk of illness [7, 10], and community approval [11, 12]. However, these studies narrowly focused on specific populations such as women or racial/ethnic minority groups or specific vaccines such as the HPV vaccine, all of which are known to have context-specific factors influencing vaccine uptake. It is not clear from prior research how these narrowly focused studies generalize into factors that impact vaccine acceptance more broadly, and how important each of these factors are in vaccination decision-making.

In addition, while the decision to vaccinate adolescents is often influenced by preferences of both adolescents and their parents, particularly among older adolescents and for HPV vaccination [13], only a small number of studies have included an adolescent perspective [14, 15]. Identifying the most influential factors in adult, adolescent, and parent vaccination decisions could help public health professionals design more effective programs to increase vaccination rates.

Increasingly, stated-preference methods are used to identify the relative importance of factors or attributes associated with health programs [16–18]. Choice experiments elicit preferences by asking respondents whether they would choose an attribute-based description of a service or program. The choices and trade-offs that respondents make can be used to infer the relative importance of each attribute and can be a powerful tool to help policy makers design programs that are aligned with patient preferences. The goal of this study was to identify the most important factors related to vaccine decision-making among U.S. adults, adolescents, and parents of adolescents (parents) using a stated preference binary choice experiment.

## Methods

### Survey development

We conducted two-hour interviews with each of four focus groups using a standardized interview guide in Boston, MA and Ann Arbor, MI in 2013 to identify attributes that could be used to create hypothetical vaccination profiles in a preference survey. To include a diversity of perspectives, one group was comprised of Spanish-speaking adults (*n* = 10, including both parents and non-parents; ages 18–64 years), one of English-speaking adults (*n* = 9), one of English-speaking adolescents (*n* = 7, ages 13–17 years), and one of parents (*n* = 6).

During the focus group interviews, participants were asked to describe their experience with vaccination, and factors that were important to them in their decision to vaccinate. At the end of the focus group, they were asked to rank factors relevant in their decision to vaccinate from most to least important. We used this ranking data from the focus groups along with relevant literature and expert opinion to identify 12 factors that impact vaccine uptake among parents, adults and adolescents (e.g., out-of-pocket cost of vaccine; Table 1) and assign 2–8 levels to each factor that represented possible values (e.g., out-of-pocket cost: $10, $25, etc.; Additional file 1: Table S1). These factors and levels were the basis for creating vaccination profiles in the preference survey.

**Table 1:** Attributes and definitions for adult respondents ^a^

Attribute	Definition
Seriousness of illness	This is how bad or serious the illness would be for you if you got sick.
Duration of illness	This is how long you would have symptoms of the illness if you got sick.
Vaccine effectiveness	This is the likelihood that you will NOT get the illness that the vaccine can prevent if you are vaccinated. Vaccines with greater effectiveness are better at reducing your risk of illness and death.
Your risk of illness without vaccination	This is the likelihood you will get the illness that the vaccine can prevent if you are not vaccinated.
Your risk of death without vaccination	This is the likelihood you will die from the illness that the vaccine can prevent if you are not vaccinated.
Your risk of severe side effect from vaccination	This is the likelihood that after vaccination, you will get a serious long-term or permanent disability that affects your nervous system. You would no longer be able to do daily activities because of the side effect. The side effect is caused by what is in the vaccine.
Length of time vaccine has been available	This is the number of years that the vaccine has been available to the public in the United States.
Location	This is the place you go to get vaccinated.
Time	This is the total time it would take you to get one shot of the vaccine, including: (1) Time you wait (2) Time you spend with the health care professional (3) Travel time to and from the location where you get the vaccine.
Type of health care professional giving vaccine	This is the type of healthcare professional who gives you the vaccine.
Primary care provider recommendation	This is the recommendation of your primary care provider about getting the vaccine. A primary care provider is the person you see for regular medical care and can be a doctor, a nurse practitioner (NP) or a physician assistant (PA).
Cost after insurance	This is the amount that your family would pay for you to receive the vaccine. This amount would not be covered by insurance. You would be paying with money that you have available today.

^a^Attributes were the same for adolescents and parents, but definitions differed slightly. Additional attributes included risk of illness with vaccination and risk of death with vaccination which were derived by multiplying the risk of illness/death without vaccination by the vaccine effectiveness. These additional attributes and their levels are available in Additional file 2: Table S2

**Fig. 1:**
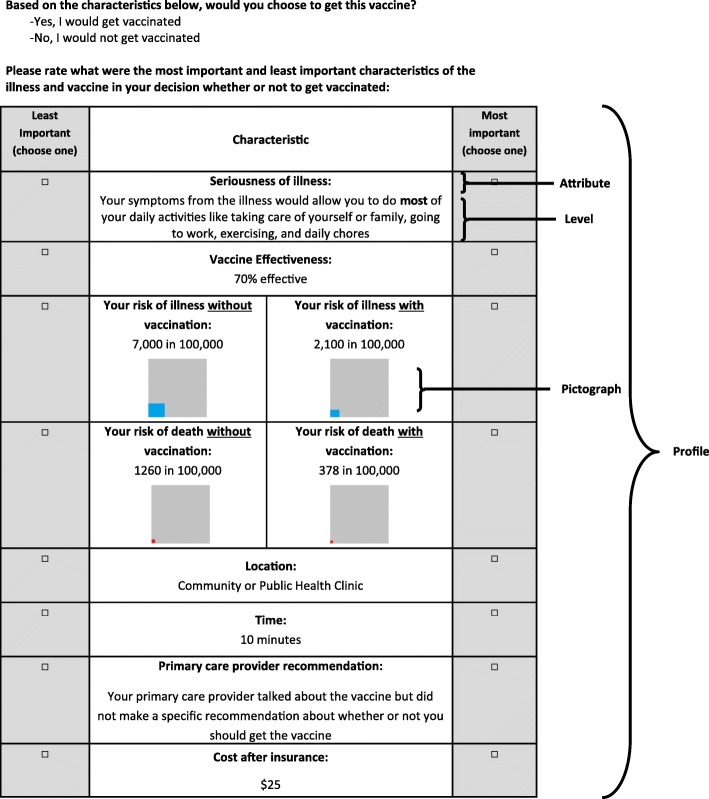
Sample conjoint analysis question for adult respondent

Levels were chosen to represent meaningful differences within attributes, with a range of values that were relevant for current or anticipated future vaccine decision-making scenarios based on our literature review and expert opinion. Further adjustments were made to the levels during pretesting if respondents indicated that some levels were not meaningfully different. We derived two descriptive attributes (risk of illness with vaccination, risk of death with vaccination; Additional file 2: Table S2) with 16 levels in each to help respondents understand how the risk of illness and death would change with vaccination, depending on the effectiveness of the vaccine (included in the survey as 20%, 70%, 95%, and 99% relative risk reduction). Risk of illness with vaccination was derived by multiplying the risk of illness without vaccination by the vaccine effectiveness. Risk of death with vaccination was derived by multiplying the risk of death without vaccination by the vaccine effectiveness. The use of visual aids has been strongly recommended for experiments of this type [19]. Therefore, pictographs were created to improve respondent comprehension of risk information with and without vaccination (Fig. 1) [20].

We developed three versions of a vaccine preference survey, one each for adults, adolescents, and parents. Attributes were the same across the survey versions, but the wording of definitions was adjusted as appropriate (e.g., school for adolescents versus work for adults). The survey included an introduction to the 12 attributes, their definitions, and levels, as well as a practice question to orient the respondent to the task. The respondent was then presented with 5 hypothetical vaccine partial profiles with 8 of the 12 attributes in each.

For each of the 5 profiles that they saw, respondents were asked a binary choice question and a best-worst scaling question. We chose for respondents to see one profile at a time to allow us to use both of these methods together. The binary choice (yes/no) question came before each profile; we asked adult and adolescent respondents about whether they would choose to get a vaccine for themselves (Fig. 1). Parents were asked about vaccinating their adolescent child. Also, in each profile, respondents were asked to choose the most important and least important factors in their decision to receive or not receive the vaccine (Fig. 1). Only data from the binary choice question were used for this analysis.

The partial profile design always included the “vaccine effectiveness” attribute in all profiles with randomly assigned levels. Of the remaining 11 attributes, we chose 7 to vary in each profile. The process of identifying the 7 to vary in each profile was based on a fractional factorial design [21] that ensured attribute balance and statistical efficiency. [22] This design ensures the best orthogonal combination of attribute levels to minimize correlation between attributes and levels. We evaluated the performance of the design with the D-efficiency statistic, which provides a means of evaluating the most efficient combination of choice sets based on balance and orthogonality [23]. Sixty-five profiles were created in this process. Divided into 13 blocks of 5 profiles, and each respondent was randomized to one of these blocks. Prior to survey administration, we manually screened all profiles for logical consistency.

At the end of the survey, we collected respondents’ health insurance status, general health status, confidence in their responses, and attitudes and behaviors concerning vaccines (Table 2). The online survey vendor also collected respondents’ demographic information and whether they had Internet access prior to joining the research panel, which is an indication of otherwise unmeasured socio-economic factors.

**Table 2:** Respondent demographics, confidence in responses, and vaccination practices and beliefs

	Adults (*n* = 334) n (%)	Adolescents (*n* = 316) n (%)	Parents (*n* = 339) n (%)
Gender			
Female	153 (45.8)	171 (54.1)	161 (47.5)
Age			
13–15	0	149 (47.2)	0
16–17	0	167 (52.9)	0
18–29	63 (18.9)	0	5 (1.5)
30–49	133 (39.8)	0	234 (69.0)
50–64	138 (41.3)	0	100 (29.5)
Education			
Less than high school	19 (5.7)	316 (100)	17 (5.0)
High school/Some college	183 (54.8)	0	161 (47.5)
Bachelor’s degree or higher	132 (39.5)	0	161 (47.5)
Race/Ethnicity			
White, non-Hispanic	255 (76.4)	169 (53.5)	247 (72.9)
Black, non-Hispanic	23 (6.9)	30 (9.5)	18 (5.3)
Other, non-Hispanic	27 (8.1)	43 (13.6)	25 (7.4)
Hispanic	7 (2.1)	74 (23.4)	49 (14.6)
Region			
Northeast	64 (19.2)	30 (9.5)	65 (19.2)
Midwest	83 (24.9)	91 (28.8)	88 (26.0)
South	119 (35.6)	112 (35.4)	120 (35.4)
West	68 (20.4)	83 (26.3)	66 (19.5)
Household income			
< $25,000	59 (17.7)	63 (19.9)	28 (8.3)
$25,000–$49,999	56 (16.8)	75 (23.7)	43 (12.7)
$50,000–$99,999	112 (33.6)	108 (34.1)	129 (38.1)
> $100,000	107 (32.1)	70 (22.1)	139 (41.0)
Marital Status			
Divorced/Separated/Widowed	51 (15.3)	0	31 (9.2)
Married/Living with partner	198 (59.3)	0	301 (88.8)
Never married	85 (25.5)	316 (100)	7 (2.1)
Employment Status			
Employed	234 (70.1)	NA	261 (77.0)
Not employed	100 (29.9)		78 (22.9)
Insurance			
Private	233 (71.0)	136 (43.3)	262 (78.4)
Medicaid	53 (16.1)	76 (24.2)	58 (17.4)
Have insurance, unsure of type	8 (2.4)	82 (26.1)	4 (1.2)
Do not have insurance	19 (5.8)	9 (2.9)	3 (0.9)
Other	15 (4.6)	11 (3.5)	7 (2.1)
Confidence in conjoint survey questions			
Very/somewhat confident	297 (89.5)	279 (88.6)	311 (92.6)
Not confident/guess	35 (10.5)	36 (11.4)	25 (7.4)
In general, would you say your/ your child’s health is			
Excellent/very good	177 (53.3)	240 (76.7)	302 (89.6)
Good	118 (35.5)	56 (17.9)	28 (8.3)
Fair/Poor	37 (11.1)	17 (5.4)	7 (2.1)
I believe it is important to be/to have my child vaccinated			
Always/mostly agree	195 (58.7)	215 (68.7)	276 (81.9)
Sometimes agree, sometimes disagree	99 (29.8)	74 (23.6)	46 (13.7)
Always/mostly disagree	38 (11.5)	24 (7.7)	15 (4.5)
I believe the benefits of vaccines outweigh risks			
Always/mostly agree	192 (58.5)	188 (61.0)	252 (75.5)
Sometimes agree, sometimes disagree	108 (32.9)	96 (31.1)	70 (21.0)
Always/mostly disagree	28 (8.5)	24 (7.8)	12 (3.4)
Last time you/your child was vaccinated			
< 1 year ago	158 (47.3)	157 (49.7)	173 (51.0)
1–5 years ago	85 (25.5)	129 (40.8)	145 (42.8)
> 5 years ago	85 (25.5)	29 (9.2)	18 (5.3)
Refused	6 (1.8)	1 (0.3)	3 (0.9)

Note: Numbers may not add up to the total n for the respondent group as participants could decline to answer question

### Survey administration

Prior to fielding, we conducted cognitive pretests with adults, adolescents, and parents (*n* = 38) to ensure respondents understood the survey questions as intended, and that derived attributes and visualizations were effective in conveying the relationship between vaccine effectiveness and risks of illness and death with and without vaccination. We also used these pretests to determine whether profiles could be presented to respondents with all 12 attributes. This process highlighted the cognitive burden of considering 12 attributes at once, and therefore we created partial profiles with 8 attributes. We ensured that participants could answer 5 choice questions without cognitive difficulty, and in the timeframe acceptable for our survey. We conducted a national pilot of the survey (*n* = 150) in October 2015 (data not shown).

In November 2015, we administered the final survey to a national sample of 652 adults, 1516 adolescents, and 1030 parents using KnowledgePanel (GfK Custom Research, LLC, New York, NY), a probability-based online research panel [24]. KnowledgePanel members receive a small cash incentive for each survey they complete and computer and Internet access, if needed, while participating in surveys. Adults who were also the parent of an adolescent were eligible for either the adult or the parent survey but could only be chosen once to answer in either capacity.

### Analysis

Using the binary choice data, we used logistic regression to calculate the odds that a respondent would choose to receive (or have their adolescent child receive) the vaccine described in the profile. The dependent variable was whether the respondent chose to accept or decline the vaccine in the profile, and each factor-level seen in that profile was coded as an independent variable using effect coding. With effect coding the estimated independent effects for each attribute level, including the reference level, represent the incremental effect over the mean for all respondents [25]. We controlled for respondent characteristics including respondent gender, age, U.S. region, race/ethnicity, household income, education level, employment (adults and parents only), and previous experience with a vaccine side effect.

The resulting log odds parameter estimates for each attribute-level in the model represent the relative strength of the respondent’s preference of that level compared to the mean effect. Positive levels that are significantly greater than zero indicate that respondents were more likely to choose a profile with that level relative to the mean, and negative levels that are significantly lower than zero indicate that respondents were less likely to choose a profile with that level relative to the mean. Confidence intervals (CIs) for parameter estimates that include zero indicate no difference from the mean effect at the 5% significance level.

If the CIs for levels within an attribute do not overlap, the levels are statistically different (*p* < 0.05) from each other. A significant difference between attribute-level coefficients can be interpreted as the change in preference for vaccination between these two levels, conditional on the other attribute-levels included in the survey.

We clustered standard errors using generalized estimating equations to adjust for multiple answers per respondent [26]. We stratified analyses by respondent type (adult, adolescent, or parent). We reviewed how predicted probabilities of uptake varied under different scenarios.

In sensitivity analyses we excluded: 1) respondents who reported they were not confident in their responses and 2) those that always accepted or always declined the vaccine profile presented to them. In another sensitivity analysis we include out-of-pocket cost in our model as a continuous variable instead of a categorical variable. We also explored two alternative model specifications that included terms for the absolute risk reduction due to vaccination in each profile (baseline risk *x* vaccine effectiveness). In the first model we added interaction terms to the model between vaccine effectiveness, and risk of illness and death (Additional file 7: Table S7). In the second model we added two calculated parameters for the absolute risk reduction of illness and death with vaccination and removed variables for vaccine effectiveness and baseline risk of illness and death from the model specification (Additional file 7: Table S7).

All analyses were performed in 2016 using STATA v13 (College Station, TX). The study was approved by the University of Michigan Health System Institutional Review Board.

## Results

### Sample

We analyzed completed surveys from 334 (51%) adults, 316 (21%) adolescents, and 339 (33%) parents. Respondents were generally representative of the U.S. population, with the exceptions that adults surveyed were more likely to be college educated, white, and married, adolescents were more likely to be Hispanic and lower income, and parents were more likely to be educated and white, compared to Census averages (Table 2, additional demographic information is given in Additional file 3: Table S3). Fifty three percent of adults, 77% of adolescents, and 90% of parents reported being in excellent or very good health. Approximately half of respondents in each group reported having received a vaccination within the past year.

### Choice to accept or decline vaccination

**Fig. 2:**
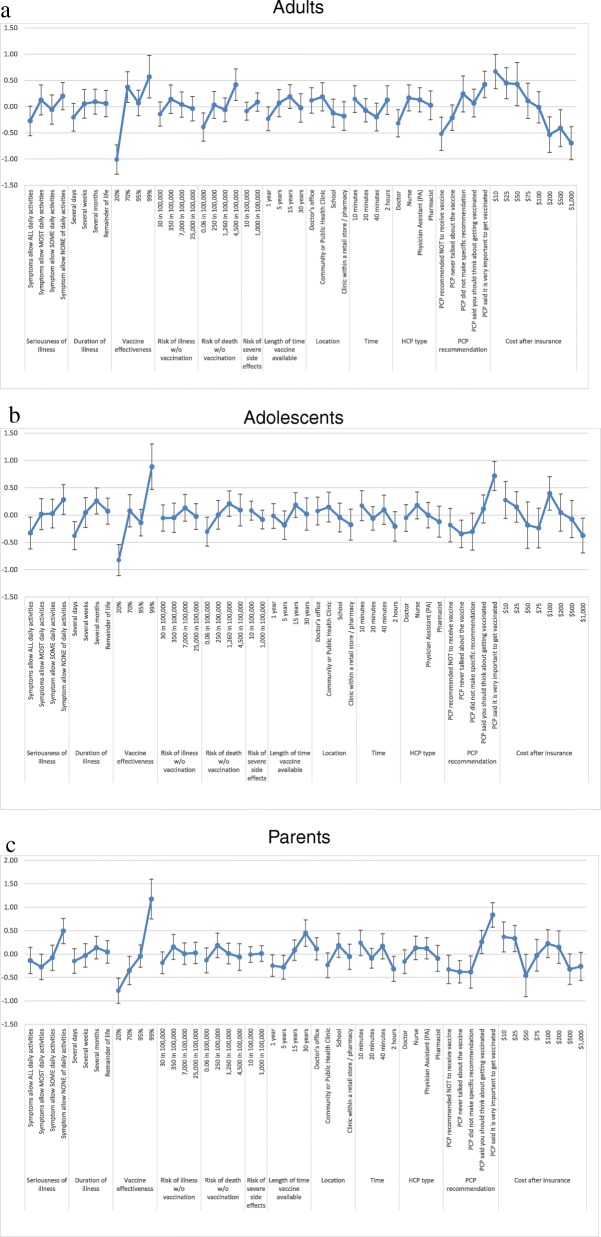
Log odds for selecting each factor, for each group

Among adults, adolescents, and parents, the choice of whether to accept or decline the vaccine presented in the profile was primarily driven by three factors: vaccine effectiveness, primary care provider (PCP) recommendation, and the out-of-pocket cost of the vaccine (Fig. 2a-c; Additional file 4: Table S4). Vaccine effectiveness was the most influential factor for all groups. For adults, there was a statistically significant increase in preference between vaccines that offered 20% effectiveness, which respondents were less likely to choose, and vaccines that offered 70% effectiveness, which respondents were more likely to choose (Fig. 2a). Gains in effectiveness above 70% did not significantly increase respondent preferences. For adolescents, there was a statistically significant increase in preference between vaccines with 20% effectiveness, which they were less likely to choose, and those with 99% effectiveness, which adolescents were more likely to choose (Fig. 2b). Vaccine effectiveness of 70% or 95% effectiveness did not impact adolescent choice. Parents were less likely to choose vaccines with 20% and 70% effectiveness, and more likely to choose vaccines with 99% effectiveness, but were indifferent to vaccines with 95% effectiveness (Fig. 2c).

Among adults, the next most influential factor in their decision to vaccinate following vaccine effectiveness was out-of-pocket cost. The large significant decrease in preference between vaccines that cost $10 at the lowest level and $1000 at the highest level was primary influenced by a statistically significant decrease in preferences between vaccines that cost $10 vs. $100 and again between vaccines that cost $100 vs. $1000 (Fig. 2a). Among adolescents and parents, preferences based on out-of-pocket cost did not follow the expected ordering; the only significant decrease in adolescent preferences came from increase in cost from $100 to $1000 (Fig. 2b), and the only significant decrease in parent preferences came from an increase in cost from $25 to $50 (Fig. 2c). Two sensitivity analyses that included cost and log cost as continuous model variables showed a statistically significant decrease in preference associated with increasing out-of-pocket cost in all groups (results not shown).

Among adolescents and parents, the next most influential factor in their decision to vaccinate following vaccine effectiveness was PCP recommendation. Adolescents were significantly more likely to accept vaccines that came with a strong PCP recommendation (“said it was very important”), and significantly less likely to accept vaccines where the PCP never talked to them about vaccines. But other positive or negative forms of recommendation did not impact respondent choice. (Fig. 2b). Parent respondents were more likely to choose a vaccine that came with either a weak (“should think about getting vaccinated”) or a strong recommendation, and a strong recommendation significantly influenced decisions over a weak recommendation. Similar to adolescents, parents were significantly less likely to accept vaccines where the PCP never talked to them about vaccines. But other types of recommendations did not impact respondent choice (Fig. 2c). Adults were significantly more likely to choose a vaccine with a strong recommendation, and less likely to choose one with a recommendation not to vaccinate, but other intermediate levels of recommendation did not significantly influence preferences.

Using our model to predict vaccine uptake based on hypothetical changes in the PCP recommendations presented in vaccine scenarios, we found that the percentage of adults who accept a vaccine is estimated to significantly increase from 47% if their PCP never talks about the vaccine, to 60% if their PCP strongly recommends the vaccine (predicted probabilities and 95% CIs in Additional file 5: Table S5). The increase in predicted uptake was even greater among the other two respondent groups. Going from a scenario without any PCP recommendation to a strong PCP recommendation, probabilities of vaccine acceptance increased from 48% to 70% among adolescents, and from 55% to 77% among parents.

Other factors besides vaccine effectiveness, out-of-pocket cost and PCP recommendation were less influential across all groups, and in many cases did not impact preferences at all (Fig. 2a-c). Adult decisions were more influenced by the risk of death without vaccination versus other groups. Adolescents and parents were more concerned than adults about the seriousness of the illness. Parents were more concerned than other groups about the length of time the vaccine had been available.

### Demographics

Respondent characteristics had mixed effects across subgroups (Table 3). There were no gender differences in adult or adolescent decisions to accept a vaccine in the survey. Among parents, mothers were less likely than fathers to accept a vaccine for their child. Non-white adolescents and parents were significantly (*p* < 0.05) more likely to accept a vaccine, compared to white respondents in those groups. Similarly, non-white adults appear more likely to accept a vaccine (*p* = 0.07). Adult respondents with a household income above $50,000 were significantly more likely (*p* = 0.01) to accept a vaccine, but adolescents above this income level were significantly less likely to accept the vaccine (*p* = 0.03) and higher income parents similarly appear less likely to accept the vaccine (*p* = 0.05). Adults and parents without side effect experience were significantly (p < 0.05) more likely to accept the vaccine presented in the survey compared with those with side effect experience. Similarly, adolescents without side effect experience appear more likely to accept a vaccine (p = 0.07). All other demographic characteristics were not significantly associated with the decision to accept the vaccine.

**Table 3:** Odds ratios for demographic variables* Indicates increased (OR > 1) or decreased (OR < 1) odds of respondent with demographic variable shown accepting a vaccine shown in a profile compared to the reference group

Attribute	Level	Adults		Adolescents		Parents of adolescents	
		OR	*P*-value	OR	*P*-value	OR	*P*-value
Respondent gender	Male	REF	–	REF	–	REF	–
	Female	0.98	0.912	1.12	0.584	**0.64†**	**0.025**
Median age category	Below median	REF	–	REF	–	REF	–
	At or greater than median age	1.11	0.595	0.67	0.055	0.70	0.073
Respondent education	< Bachelor’s degree	REF	–	–	–	REF	–
	Bachelor’s degree and higher	0.68	0.063	–	–	1.33	0.156
Region	Northeast	REF	–	REF	–	REF	–
	Midwest	1.17	0.594	1.99	0.077	0.82	0.484
	South	1.01	0.981	1.15	0.696	0.74	0.285
	West	1.07	0.837	1.45	0.339	1.33	0.349
Race/ethnicity	White, non-Hispanic	REF	–	REF	–	REF	–
	Non-white, non-Hispanic	1.54	0.068	**2.14†**	**0.001**	**2.33†**	**< 0.001**
Household income	<$50,000/yr	REF	–	REF	–	REF	–
	> = $50,000/yr	**1.71†**	**0.012**	**0.62†**	**0.028**	0.61	0.052
Employed	Yes	REF	–	–	–	REF	–
	No	1.05	0.831	–	–	0.76	0.256
Side effect experience	Yes	REF	–	REF	–	REF	–
	No	**2.33†**	**< 0.001**	1.49	0.073	**1.66†**	**0.020**

*Model also contained additional variables: Seriousness of illness, duration of illness, vaccine effectiveness, risk of illness without vaccination, risk of death without vaccination, risk of severe side effects, length of time vaccine available, location, time, healthcare provider type, PCP recommendation, and cost after insurance

**†**Indicates that odds ratio is significantly different from 1 (*p* < 0.05)

### Sensitivity analyses

More than one third of respondents were either “vaccine acceptors”, always accepting the profile presented, or “vaccine rejecters”, always rejecting the profile. Seventeen percent of adults, 24% of adolescents, and 34% of parents always accepted the profiles shown (p < 0.05 for differences across groups). Seventeen percent of adults, 14% of adolescents, and 12% of parents always rejected the profiles shown (no significant differences between groups; Additional file 6: Table S6). Sensitivity analyses that excluded these respondents, as well as those that excluded respondents who stated they were not confident in their choices (11% adults, 11% adolescents, and 7% parents), did not change our findings. Alternative model specifications that included variables for absolute risk reduction due to vaccination were not a better fit for our data (Additional file 7: Table S7).

## Discussion

Improving recommended vaccine coverage for adults and adolescents has been an ongoing public health challenge in the United States. When deciding whether to vaccinate, our results indicate that adults, adolescents, and parents generally prioritize vaccine effectiveness, out-of-pocket cost, and PCP recommendation. The out-of-pocket cost and PCP recommendation attributes are important to consider when designing vaccination programs because they have the potential to be modified through health policies.

Across all three populations, and particularly among adults, we found out-of-pocket cost to be an important predictor of whether the respondent accepted or declined the vaccine. This emphasizes the importance of programs like Vaccines for Children, and Public Health Service Section 317 discretionary funding support for adolescents and adults, respectively, which enable healthcare providers to secure vaccines at no charge and vaccinate uninsured and underinsured populations. Likewise, insurance policies can ensure that vaccines remain affordable to people of all ages and continued efforts should be made to educate the general public about the inclusion of vaccines in their health plans.

A strong PCP recommendation was shown to significantly increase vaccine acceptance in all groups, highlighting why healthcare providers should be encouraged to talk about the value of vaccines during routine patient visits. This is particularly true as we found that even a weak recommendation to vaccinate only made parents, not adults or adolescents, more likely to vaccinate. Other recommendations or lack thereof, either had no impact on preferences or influenced choices to not vaccinate. These findings underscore the need for continued training support for healthcare professionals, as they work to increase vaccine acceptability among their patients.

Adults, adolescents and parents may prioritize different factors when making decisions about vaccines. Parents were relatively more concerned about number of years the vaccine had been available. Previous research has shown that parents are more likely to refuse newer vaccines, and express concerns that newer vaccines have not been available long enough for them to trust [27, 28]. Our study indicates that parents may perceive a vaccine as being “new” and be less likely to accept it for their child for at least 5 years and may not fully trust the vaccine until it has been on the market for much longer, potentially up to 30 years. This highlights the need to educate parents and encourage physicians to discuss the safety of vaccines with parents in particular, especially for newer vaccines. Also, compared to adults, adolescents were more concerned with the seriousness and duration of the illness when making a decision to be vaccinated. Adolescents were more likely to choose vaccination if the illness lasted longer (months vs. days), or restricted them from doing their daily activities, whereas these factors did not influence adult decisions.

Previous studies on vaccine uptake have focused on specific groups, such as pregnant women [29, 30], ethnic minority and low-income groups, or specific vaccines like HPV [7, 11, 12, 31], meningococcal [32], and influenza [29] vaccines. Our results are consistent with many of these more narrowly focused studies, which found a strong association between out-of-pocket cost, vaccine effectiveness, provider recommendation, and vaccine acceptance. This study adds the strength of a relatively large national sample, a quantitative assessment of interaction between decision-making factors, and an important understanding of how the relative importance these factors vary between adults, adolescents, and parents.

This study has several limitations. Our national samples are not representative in all respects. A low response rate, particularly for the adolescent group (21% response) may impact results’ generalizability. Our analyses controlled for important variables that may impact vaccine preferences, including race/ethnicity, income and education. However, there may be unmeasured characteristics in each group that differ from national averages and may impact results. Sampling weights were not used in our analysis, as the sample sizes were too small to reliably use post-stratification weighting adjustments.

We asked respondents whether they would choose to accept a hypothetical vaccine versus no vaccine, but guidelines recommend that individuals receive numerous vaccines, with more than one vaccine often offered in one visit. Therefore, our scenarios which characterized vaccination decisions as one-off choices may not accurately describe the true decision-making context. In addition, by focusing on a hypothetical vaccine and not a particular one (e.g., HPV vaccine), we may have missed specific factors that are important in certain decision-making scenarios. We also asked respondents to imagine scenarios where they would have to pay for a vaccine at the level specified in the profile. However, most parents and adolescents do not face co-pays for recommended vaccines, so this may have impacted responses.

Additionally, we sought to understand how people prioritize between twelve factors related to vaccination decision-making, which emerged as important attributes from the focus group. However, as pre-testing highlighted the cognitive burden of considering all attributes at once, we created partial profiles with eight attributes. Partial profiles may introduce bias, although the direction of this bias is not clear, and should be considered carefully, as respondents consider their choices based on some attributes without others. In these cases where there are a large number of relevant attributes, choice experiments may not be well suited to accommodate the complexity of the decision-making process. These issues may have contributed to the lack of expected natural ordering of the preferences obtained from our results. As the field moves forward, a best-worst scaling approach could be used to first narrow down the number of attributes considered in a choice task with many attributes identified as important to the decision maker. In addition, we considered four attributes that people may collectively think of as describing the severity of illness (seriousness and duration of illness, and the risk of illness and death) separately in the survey, which may have diluted their combined importance.

And finally, the binary choice we used in our preference survey is not representative of most choice experiments, which typically ask respondents to choose between pairs of profiles in a discrete choice experiment. This binary choice format may have been underpowered to detect the importance of attribute levels in influencing choices. The sample size for the survey was determined based on a previous survey design that was revised during pre-testing, but without additional resources to increase the sample size.

In addition, the logistic regression analysis used is appropriate for these binary outcomes but does not account for random choice error which is common in choice experiments such as this one. There may also be scale effects across the adult, adolescent, and parent samples which cannot be directly evaluated in logistic regression analyses using standard approaches. The binary choice format, along with our partial profile design, also prohibited us from identifying respondents who may have answered all questions based on a particular attribute (attribute dominance, e.g., lowest cost). However, other sensitivity analyses we performed did not produce markedly different results from our base case analysis. Analyses of best-worst scaling results (not shown) indicate that these two methods are complementary and produce consistent results.

We did not build repeat profiles into the experimental design to identify respondents whose choices suggested a lack of attention or task comprehension. However, sensitivity analyses that excluded respondents who always accepted or rejected the vaccine, as well as who stated they were not confident in their choices, did not change our findings.

## Conclusions

Our study found that adults, adolescents and parents considered vaccine effectiveness, out-of-pocket cost, and PCP recommendation as the most important factors related to vaccination. They did not prioritize the risk of illness, the risk of vaccine side effects, vaccination location, or time for vaccination when choosing whether to be vaccinated.

Low out-of-pocket costs and healthcare provider recommendation may be effective levers to influence vaccine uptake. Healthcare providers should be cognizant of the strength of their recommendation and their role in increasing vaccination coverage rates among adults and adolescents. Healthcare policies and immunization programs should strengthen access to vaccines by ensuring that out-of-pocket cost is not a barrier.

## Additional files

Additional file 1: **Table S1.** Attributes and levels for adult respondents. (DOCX 18 kb)

Additional file 2: **Table S2.** Derived attributes and levels for adult respondents. (DOCX 14 kb)

Additional file 3: **Table S3.** Additional Demographics. (DOCX 18 kb)

Additional file 4: **Table S4.** Log odds of selecting each factor by age group. (DOCX 25 kb)

Additional file 5: **Table S5.** Predicted probabilities for primary care provider (PCP) recommendation. (DOCX 13 kb)

Additional file 6: **Table S6.** Percentage of respondents who always accepted or always rejected profiles. (DOCX 13 kb)

Additional file 7: **Table S7.** Log odds for alternative model specifications. (DOCX 34 kb)

## Data Availability

The datasets generated and/or analyzed during the current study are not publicly available but are available from the corresponding author upon reasonable request.

## Abbreviations

HPV: Human papillomavirus
PCP: Primary care provider
U.S: United States
