# Supplementary material for: Use of a choice survey to identify adult, adolescent and parent preferences for vaccination in the United States
Source: J Patient Rep Outcomes. 2019 Jul 29;3:51. doi: 10.1186/s41687-019-0135-0 (PMC6663948; doi:10.1186/s41687-019-0135-0)
Supplement: Supplementary file 1 — Table S1. Attributes and levels for adult respondents. (DOCX 18 kb) [file 41687_2019_135_MOESM1_ESM.docx]

Table S1. Attributes and levels for adult respondents ^*^

| **Attribute** | **Hover-Over Definition** | **Levels** |
| --- | --- | --- |
| Seriousness of illness | This is how bad or serious the illness would be for you if you got sick. | 1. Your symptoms from the illness would allow you to do **all** of your daily activities like taking care of yourself or family, going to work, exercising, and finishing regular household chores 2. Your symptoms from the illness would allow you to do **most** of your daily activities taking care of yourself or family, going to work, exercising, and finishing regular household chores 3. Your symptoms from the illness would allow you to do **some** of your daily activities like taking care of yourself or family, going to work, exercising, and finishing regular household chores 4. Your symptoms from the illness would allow you to do **none** of your daily activities like taking care of yourself or family, going to work, exercising, and finishing regular household chores |
| Duration of illness | This is how long you would have symptoms of the illness if you got sick. | 1. Several days 2. Several weeks 3. Several months 4. Remainder of life |
| Vaccine effectiveness | This is the likelihood that you will NOT get the illness that the vaccine can prevent if you are vaccinated. Vaccines with greater effectiveness are better at reducing your risk of illness and death. | 1. 20% 2. 70% 3. 95% 4. 99% |
| Your risk of illness without vaccination | This is the likelihood you will get the illness that the vaccine can prevent if you are not vaccinated. | 1. 30 in 100,000 2. 350 in 100,000 3. 7,000 in 100,000 4. 25,000 in 100,000 |
| Your risk of death without vaccination | This is the likelihood you will die from the illness that the vaccine can prevent if you are not vaccinated. | 1. 0.06 in 100,000 2. 250 in 100,000 3. 1,260 in 100,000 4. 4,500 in 100,000 |
| Your risk of severe side effect from vaccination | This is the likelihood that after vaccination, you will get a serious long-term or permanent disability that affects your nervous system. You would no longer be able to do daily activities because of the side effect. The side effect is caused by what is in the vaccine. | 1. 10 in 100,000 2. 1,000 in 100,000 |
| Length of time vaccine has been available | This is the number of years that the vaccine has been available to the public in the United States. | 1. 1 year 2. 5 years 3. 15 years 4. 30 years |
| Location | This is the place you go to get vaccinated. | 1. Doctor’s office 2. Community or Public Health Clinic 3. Work 4. Clinic within a retail store or pharmacy |
| Time | This is the **total time** it would take you to get one shot of the vaccine, including:  (1) Time you wait  (2) Time you spend with the health care professional (3) Travel time to and from the location where you get the vaccine. | 1. 10 minutes 2. 20 minutes 3. 40 minutes 4. 2 hours |
| Type of health care professional giving vaccine | This is the type of healthcare professional who gives you the vaccine. | 1. Doctor 2. Nurse 3. Physician Assistant (PA) 4. Pharmacist |
| Primary care provider recommendation | This is the recommendation of **your** primary care provider about getting the vaccine. A primary care provider is the person you see for regular medical care and can be a doctor, a nurse practitioner (NP) or a physician assistant (PA). | 1. Your primary care provider recommended that you should **not** receive the vaccine. 2. Your primary care provider never talked about the vaccine. 3. Your primary care provider talked about the vaccine but did not make a specific recommendation about whether or not you should get the vaccine. 4. Your primary care provider talked about the vaccine and said that you should think about getting vaccinated. 5. Your primary care provider talked about the vaccine and said that it was very important for to get vaccinated. |
| Cost after insurance | This is the amount that your family would pay for you to receive the vaccine. **This amount would not be covered by insurance.** You would be paying with money that you have available today. | 1. $10 2. $25 3. $50 4. $75 5. $100 6. $200 7. $500 8. $1000 |

^*^ Additional attributes included risk of illness with vaccination and risk of death with vaccination which were derived by multiplying the risk of illness/death without vaccination by the vaccine effectiveness. These additional attributes and their levels are available in Additional file 3: Table S3.
