# Supplementary material for: Use of a choice survey to identify adult, adolescent and parent preferences for vaccination in the United States
Source: J Patient Rep Outcomes. 2019 Jul 29;3:51. doi: 10.1186/s41687-019-0135-0 (PMC6663948; doi:10.1186/s41687-019-0135-0)
Supplement: Supplementary file 2 — Table S2. Derived attributes and levels for adult respondents. (DOCX 14 kb) [file 41687_2019_135_MOESM2_ESM.docx]

Table S2. Derived attributes and levels

| **Attribute** | **Hover-Over Definition** | **Levels** |
| --- | --- | --- |
| Your risk of illness with vaccination ^a^ | This is the likelihood you will get the illness that the vaccine can prevent if you are vaccinated. | 1. 0.3 in 100,000  2. 1.5 in 100,000  3. 3.5 in 100,000  4. 9 in 100,000  5. 17.5 in 100,000  6. 24 in 100,000  7. 70 in 100,000  8. 105 in 100,000  9. 250 in 100,000  10. 280 in 100,000  11. 350 in 100,000  12. 1,250 in 100,000  13. 2,100 in 100,000  14. 5,600 in 100,000  15. 7,500 in 100,000  16. 20,000 in 100,000 |
| Your risk of death with vaccination ^b^ | This is the likelihood you will die from the illness that the vaccine can prevent if you are vaccinated | 1. 0.0006 in 100,000  2. 0.003 in 100,000  3. 0.018 in 100,000  4. 0.048 in 100,000  5. 2.5 in 100,000  6. 12.5 in 100,000  7. 12.6 in 100,000  8. 45 in 100,000  9. 63 in 100,000  10. 75 in 100,000  11. 200 in 100,000  12. 225 in 100,000  13. 378 in 100,000  14. 1,008 in 100,000  15. 1,350 in 100,000  16. 3,600 in 100,000 |

^a^ Risk of illness with vaccination was derived by multiplying the risk of illness without vaccination by the vaccine effectiveness

^b^ Risk of death with vaccination was derived by multiplying the risk of death without vaccination by the vaccine effectiveness
