# Supplementary material for: Use of a choice survey to identify adult, adolescent and parent preferences for vaccination in the United States
Source: J Patient Rep Outcomes. 2019 Jul 29;3:51. doi: 10.1186/s41687-019-0135-0 (PMC6663948; doi:10.1186/s41687-019-0135-0)
Supplement: Supplementary file 3 — Table S3. Additional Demographics. (DOCX 18 kb) [file 41687_2019_135_MOESM3_ESM.docx]

Table S3. Additional Demographics

|  | **Adults**  (n=334)  n (%) | **Adolescents**  (n=316)  n (%) | **Parents of Adolescents** (n=339)  n (%) |
| --- | --- | --- | --- |
| **Internet access** |  |  |  |
| Yes | 270 (80.8) | 312 (98.7) | 331 (97.6) |
| **I believe I have a social responsibility to family/friends to help protect them from illness by being/have my child be vaccinated** |  |  |  |
| Always/mostly agree | 203 (61.1) | 210 (67.1) | 248 (73.6) |
| Sometimes agree, sometimes disagree | 85 (25.6) | 73 (23.3) | 57 (16.9) |
| Always/mostly disagree | 44 (13.3) | 30 (9.6) | 32 (9.5) |
| **Reason no vaccine in last year ^a^** |  |  |  |
| Up-to-date on vaccinations | 116 (73.4) | 116 (73.4) | 142 (87.1) |
| Concerns about vaccine safety | 15 (9.5) | 15 (9.5) | 10 (6.1) |
| Did not know enough about vaccine | 13 (8.2) | 13 (8.2) | 3 (1.8) |
| Other | 15 (9.5) | 15 (9.5) | 9 (5.5) |
| **Consider vaccination in the future** |  |  |  |
| Yes | 247 (75.1) | 256 (81.3) | 297 (88.1) |
| No | 29 (8.8) | 14 (4.4) | 6 (1.8) |
| Don’t know | 53 (16.1) | 45 (14.3) | 34 (10.1) |
| **Have you/your child or anyone in your family ever experienced a side effect of vaccination** |  |  |  |
| Yes | 73 (22.1) | 76 (24.2) | 73 (21.7) |
| No | 189 (57.1) | 156 (49.7) | 240 (71.4) |
| Don’t know | 69 (20.9) | 82 (26.1) | 23 (6.9) |
| **Have you/your child or anyone in your family ever had a disease vaccines can prevent** |  |  |  |
| Yes | 83 (25.5) | 78 (24.8) | 57 (17.0) |
| No | 243 (74.5) | 236 (75.2) | 279 (83.0) |
| **Do you/ your child have a place you usually go when you need routine medical care** |  |  |  |
| Yes | 272 (82.2) | 280 (88.9) | 323 (95.9) |
| No | 43 (13.0) | 14 (4.4) | 14 (4.2) |
| Don’t know | 16 (4.8) | 21 (6.7) | 0 |
| **I trust the info I receive from my/my child’s PCP about vaccines** |  |  |  |
| Always/mostly agree | 237 (87.1) | 247 (88.5) | 292 (91.3) |
| Sometimes agree, sometimes disagree | 28 (10.3) | 30 (10.8) | 23 (7.2) |
| Always/mostly disagree | 7 (2.6) | 2 (0.7) | 5 (1.6) |
| **Are you/ your child at high risk for getting a vaccine-preventable illness?** |  |  |  |
| Yes | 41 (12.4) | 32 (10.3) | 29 (8.7) |
| No | 222 (67.3) | 195 (62.7) | 258 (77.3) |
| Don’t know | 67 (20.3) | 84 (27.0) | 47 (14.1) |
| **Thinking of particular vaccine or illness** |  |  |  |
| Yes | 67 (20.4) | 77 (24.6) | 60 (17.9) |
| No | 261 (79.6) | 236 (75.4) | 275 (82.1) |

^a^ Among those who had not received a vaccination in the past year
