# Supplementary material for: Use of a choice survey to identify adult, adolescent and parent preferences for vaccination in the United States
Source: J Patient Rep Outcomes. 2019 Jul 29;3:51. doi: 10.1186/s41687-019-0135-0 (PMC6663948; doi:10.1186/s41687-019-0135-0)
Supplement: Supplementary file 4 — Table S4. Log odds of selecting each factor by age group. (DOCX 25 kb) [file 41687_2019_135_MOESM4_ESM.docx]

Table S4. Log odds model coefficients: relative preference for each attribute-level compared to the mean effect*

| Attribute | Level | Adults | | Adolescents | | Parents of adolescents | |
| --- | --- | --- | --- | --- | --- | --- | --- |
|  |  | log odds | 95% CI | log odds | 95% CI | log odds | 95% CI |
| Seriousness of illness | Symptom would allow ALL of your daily activities | -0.27 | -0.55 , 0.01 | **-0.33†** | **-0.62 , -0.04** | -0.14 | -0.42 , 0.14 |
|  | Symptom would allow MOST of your daily activities | 0.13 | -0.16 , 0.41 | 0.02 | -0.27 , 0.30 | **-0.27†** | **-0.55 , 0.00** |
|  | Symptom would allow SOME of your daily activities | -0.06 | -0.33 , 0.22 | 0.03 | -0.23 , 0.29 | -0.08 | -0.35 , 0.19 |
|  | Symptom would allow NONE of your daily activities | 0.20 | -0.06 , 0.46 | **0.28†** | **0.01 , 0.56** | **0.49†** | **0.22 , 0.76** |
| Duration of illness | Several days | -0.20 | -0.47 , 0.06 | **-0.38†** | **-0.63 , -0.12** | -0.15 | -0.42 , 0.11 |
|  | Several weeks | 0.05 | -0.22 , 0.33 | 0.05 | -0.23 , 0.32 | -0.03 | -0.28 , 0.22 |
|  | Several months | 0.09 | -0.14 , 0.33 | **0.26†** | **0.02 , 0.50** | 0.14 | -0.10 , 0.37 |
|  | Remainder of life | 0.06 | -0.19 , 0.31 | 0.07 | -0.17 , 0.31 | 0.05 | -0.19 , 0.28 |
| Vaccine effectiveness | 20% | **-1.01†** | **-1.29 , -0.73** | **-0.82†** | **-1.11 , -0.54** | **-0.78†** | **-1.05 , -0.51** |
|  | 70% | **0.37†** | **0.08 , 0.66** | 0.08 | -0.22 , 0.37 | **-0.35†** | **-0.65 , -0.05** |
|  | 95% | 0.07 | -0.17 , 0.31 | -0.14 | -0.38 , 0.10 | -0.04 | -0.28 , 0.19 |
|  | 99% | **0.57†** | **0.16 , 0.97** | **0.88†** | **0.47 , 1.30** | **1.18†** | **0.75 , 1.60** |
| Risk of illness without vaccination | 30 in 100,000 | -0.14 | -0.38 , 0.09 | -0.06 | -0.30 , 0.18 | -0.19 | -0.42 , 0.05 |
|  | 350 in 100,000 | 0.14 | -0.13 , 0.41 | -0.05 | -0.32 , 0.22 | 0.15 | -0.11 , 0.42 |
|  | 7,000 in 100,000 | 0.04 | -0.20 , 0.28 | 0.13 | -0.11 , 0.38 | 0.01 | -0.22 , 0.24 |
|  | 25,000 in 100,000 | -0.04 | -0.27 , 0.19 | -0.03 | -0.26 , 0.21 | 0.02 | -0.21 , 0.26 |
| Risk of death without vaccination | 0.06 in 100,000 | **-0.39†** | **-0.66 , -0.12** | **-0.30†** | **-0.57 , -0.04** | -0.13 | -0.40 , 0.13 |
|  | 250 in 100,000 | 0.03 | -0.23 , 0.29 | 0.01 | -0.26 , 0.27 | 0.18 | -0.08 , 0.44 |
|  | 1,260 in 100,000 | -0.06 | -0.29 , 0.17 | 0.21 | -0.03 , 0.44 | 0.01 | -0.21 , 0.23 |
|  | 4,500 in 100,000 | **0.42†** | **0.12 , 0.72** | 0.09 | -0.20 , 0.38 | -0.06 | -0.35 , 0.22 |
| Risk of severe side effects | 10 in 100,000 | -0.09 | -0.26 , 0.09 | 0.08 | -0.09 , 0.26 | -0.01 | -0.18 , 0.16 |
|  | 1,000 in 100,000 | 0.09 | -0.09 , 0.26 | -0.08 | -0.26 , 0.09 | 0.01 | -0.16 , 0.18 |
| Length of time vaccine available | 1 year | **-0.23†** | **-0.46 , -0.01** | -0.02 | -0.24 , 0.21 | **-0.25†** | **-0.48 , -0.02** |
|  | 5 years | 0.07 | -0.19 , 0.33 | -0.18 | -0.44 , 0.08 | **-0.28†** | **-0.54 , -0.03** |
|  | 15 years | 0.19 | -0.04 , 0.42 | 0.18 | -0.04 , 0.41 | 0.08 | -0.13 , 0.30 |
|  | 30 years | -0.03 | -0.30 , 0.25 | 0.02 | -0.28 , 0.31 | **0.45†** | **0.16 , 0.73** |
| Location | Doctor's office | 0.12 | -0.13 , 0.36 | 0.07 | -0.18 , 0.32 | 0.11 | -0.13 , 0.35 |
|  | Community or Public Health Clinic | 0.19 | -0.08 , 0.46 | 0.15 | -0.12 , 0.42 | -0.24 | -0.50 , 0.03 |
|  | School | -0.12 | -0.39 , 0.14 | -0.04 | -0.31 , 0.22 | 0.18 | -0.07 , 0.44 |
|  | Clinic within a retail store / pharmacy | -0.18 | -0.46 , 0.09 | -0.18 | -0.46 , 0.11 | -0.06 | -0.33 , 0.21 |
| Time | 10 minutes | 0.14 | -0.11 , 0.40 | 0.17 | -0.10 , 0.45 | 0.24 | -0.03 , 0.51 |
|  | 20 minutes | -0.07 | -0.29 , 0.15 | -0.06 | -0.28 , 0.15 | -0.09 | -0.30 , 0.12 |
|  | 40 minutes | -0.20 | -0.46 , 0.06 | 0.10 | -0.17 , 0.36 | 0.17 | -0.10 , 0.44 |
|  | 2 hours | 0.13 | -0.15 , 0.40 | -0.21 | -0.48 , 0.06 | **-0.32†** | **-0.59 , -0.05** |
| Healthcare provider type | Doctor | **-0.32†** | **-0.58 , -0.06** | -0.05 | -0.30 , 0.19 | -0.16 | -0.41 , 0.09 |
|  | Nurse | 0.17 | -0.08 , 0.41 | 0.18 | -0.07 , 0.42 | 0.13 | -0.12 , 0.38 |
|  | Physician Assistant (PA) | 0.13 | -0.10 , 0.36 | 0.00 | -0.24 , 0.23 | 0.12 | -0.10 , 0.35 |
|  | Pharmacist | 0.03 | -0.25 , 0.30 | -0.12 | -0.41 , 0.16 | -0.09 | -0.37 , 0.18 |
| PCP recommendation | PCP recommended NOT to receive vaccine | **-0.52†** | **-0.84 , -0.20** | -0.18 | -0.49 , 0.12 | **-0.33†** | **-0.63 , -0.03** |
|  | PCP never talked about the vaccine | -0.21 | -0.45 , 0.03 | **-0.34†** | **-0.60 , -0.09** | **-0.38†** | **-0.62 , -0.14** |
|  | PCP did not make specific recommendation | 0.24 | -0.10 , 0.59 | -0.30 | -0.64 , 0.04 | **-0.38†** | **-0.73 , -0.04** |
|  | PCP said should think about getting vaccinated | 0.07 | -0.20 , 0.33 | 0.11 | -0.14 , 0.37 | **0.26†** | **0.01 , 0.51** |
|  | PCP said it is very important to get vaccinated | **0.42†** | **0.17 , 0.67** | **0.72†** | **0.45 , 0.98** | **0.83†** | **0.57 , 1.10** |
| Cost after insurance | $10 | **0.67†** | **0.34 , 1.00** | 0.28 | -0.06 , 0.61 | **0.37†** | **0.05 , 0.69** |
|  | $25 | **0.45†** | **0.15 , 0.74** | 0.15 | -0.13 , 0.43 | **0.33†** | **0.05 , 0.61** |
|  | $50 | **0.43†** | **0.02 , 0.84** | -0.18 | -0.61 , 0.24 | **-0.46†** | **-0.91 , -0.01** |
|  | $75 | 0.11 | -0.23 , 0.45 | -0.24 | -0.60 , 0.13 | -0.03 | -0.37 , 0.31 |
|  | $100 | -0.01 | -0.31 , 0.28 | **0.40†** | **0.09 , 0.70** | 0.22 | -0.08 , 0.52 |
|  | $200 | **-0.53†** | **-0.87 , -0.20** | 0.05 | -0.30 , 0.39 | 0.15 | -0.20 , 0.49 |
|  | $500 | **-0.41†** | **-0.76 , -0.06** | -0.07 | -0.41 , 0.27 | -0.32 | -0.65 , 0.00 |
|  | $1,000 | **-0.70†** | **-1.01 , -0.38** | **-0.37†** | **-0.69 , -0.06** | -0.26 | -0.56 , 0.03 |
| Respondent gender | Male | 0.06 | -0.11 , 0.23 | -0.03 | -0.20 , 0.15 | 0.18 | -0.01 , 0.37 |
|  | Female | -0.06 | -0.23 , 0.11 | 0.03 | -0.15 , 0.20 | -0.18 | -0.37 , 0.01 |
| Median age category | Below median | -0.08 | -0.25 , 0.09 | 0.15 | -0.02 , 0.33 | 0.05 | -0.14 , 0.23 |
|  | At or greater than median age | 0.08 | -0.09 , 0.25 | -0.15 | -0.33 , 0.02 | -0.05 | -0.23 , 0.14 |
| Respondent education | < Bachelor’s degree | 0.14 | -0.04 , 0.32 | - | - | -0.11 | -0.30 , 0.08 |
|  | Bachelor’s degree and higher | -0.14 | -0.32 , 0.04 | - | - | 0.11 | -0.08 , 0.30 |
| Region | Northeast | -0.09 | -0.40 , 0.23 | -0.29 | -0.74 , 0.17 | -0.09 | -0.43 , 0.26 |
|  | Midwest | 0.06 | -0.23 , 0.36 | 0.30 | -0.01 , 0.62 | -0.02 | -0.33 , 0.30 |
|  | South | -0.10 | -0.36 , 0.17 | -0.09 | -0.37 , 0.20 | -0.16 | -0.45 , 0.13 |
|  | West | 0.12 | -0.21 , 0.44 | 0.07 | -0.24 , 0.38 | 0.26 | -0.09 , 0.61 |
| Race/ethnicity | White, non-Hispanic | -0.20 | -0.40 , 0.01 | **-0.33†** | **-0.52 , -0.15** | **-0.43†** | **-0.65 , -0.21** |
|  | Non-white, non-Hispanic | 0.20 | -0.01 , 0.40 | **0.33†** | **0.15 , 0.52** | **0.43†** | **0.21 , 0.65** |
| Household income | <$50,000/yr | **-0.22†** | **-0.41 , -0.04** | 0.09 | -0.09 , 0.27 | 0.24 | 0.00 , 0.48 |
|  | >=$50,000/yr | **0.22†** | **0.04 , 0.41** | -0.09 | -0.27, 0.09 | -0.24 | -0.48 , 0.00 |
| Employed | Yes | -0.03 | -0.23 , 0.16 | - | - | 0.17 | -0.06 , 0.40 |
|  | No | 0.03 | -0.16 , 0.23 | - | - | -0.17 | -0.40 , 0.06 |
| Side effect experience | Yes | **-0.41†** | **-0.62 , -0.21** | -0.26**†** | -0.47 , -0.05 | **-0.29†** | **-0.51 , -0.08** |
|  | No | **0.41†** | **0.21 , 0.62** | 0.26**†** | 0.05 , 0.47 | **0.29†** | **0.08 , 0.51** |

*Odds ratios for demographic variables are presented in Additional file 6: Table S6

**†** Indicates that the coefficient is significantly greater than zero (p<0.05). Positive levels that are significantly greater than zero indicate that respondents were more likely to choose a profile with that level relative to the mean, and negative levels that are significantly lower than zero indicate that respondents were less likely to choose a profile with that level relative to the mean.
