# Supplementary material for: Use of a choice survey to identify adult, adolescent and parent preferences for vaccination in the United States
Source: J Patient Rep Outcomes. 2019 Jul 29;3:51. doi: 10.1186/s41687-019-0135-0 (PMC6663948; doi:10.1186/s41687-019-0135-0)
Supplement: Supplementary file 5 — Table S5. Predicted probabilities for primary care provider (PCP) recommendation. (DOCX 13 kb) [file 41687_2019_135_MOESM5_ESM.docx]

Table S5. Predicted probabilities for primary care provider (PCP) recommendation (95% confidence interval)*

| Attribute description | Adult | Adolescent | Parent of adolescent |
| --- | --- | --- | --- |
| PCP never talked about the vaccine | 46.5%  (40.3% - 52.6%) | 48.1%  (41.1% - 54.8%) | 54.8%  (48.7% - 60.9%) |
| PCP said it is very important to get vaccinated | 59.5%  (53.5% - 65.6%) | 69.8%  (63.9% - 75.6%) | 77.0%  (72.0% - 82.0%) |

*Predictions were created with all other attributes were set to their actual value in the data set.
