# Supplementary material for: Use of a choice survey to identify adult, adolescent and parent preferences for vaccination in the United States
Source: J Patient Rep Outcomes. 2019 Jul 29;3:51. doi: 10.1186/s41687-019-0135-0 (PMC6663948; doi:10.1186/s41687-019-0135-0)
Supplement: Supplementary file 6 — Table S6. Percentage of respondents who always accepted or always rejected profiles. (DOCX 13 kb) [file 41687_2019_135_MOESM6_ESM.docx]

Table S6. Percentage of respondents who always accepted or always rejected profiles*

|  | Accepted all profiles | Rejected all profiles |
| --- | --- | --- |
| All groups (n=989) | 25.3% | 14.1% |
| Adults (n=334) | 17.4% | 16.5% |
| Adolescents (n=316) | 24.4% | 14.2% |
| Parents of adolescents (n=339) | 33.9% | 11.5% |

* The proportion of respondents who accepted or rejected all profiles is significantly different across subgroups (chi square, p<0.0001)
