# Supplementary material for: Use of a choice survey to identify adult, adolescent and parent preferences for vaccination in the United States
Source: J Patient Rep Outcomes. 2019 Jul 29;3:51. doi: 10.1186/s41687-019-0135-0 (PMC6663948; doi:10.1186/s41687-019-0135-0)
Supplement: Supplementary file 7 — Table S7. Log odds for alternative model specifications. (DOCX 34 kb) [file 41687_2019_135_MOESM7_ESM.docx]

Table S7. Log odds for alternative model specifications

|  |  | Model 1:  Risk of Illness x VE  Risk of Death x VE | | | Model 2:  Risk of Illness x VE  Risk of Death x VE  Risk of illness and death, VE removed | | |
| --- | --- | --- | --- | --- | --- | --- | --- |
| Attribute | Level | Adults | Adolescents | Parents of adolescents | Adults | Adolescents | Parents of adolescents |
| Seriousness of illness | Symptom would allow ALL of your daily activities | **-1.48*** | -0.59 | -0.24 | **-0.51*** | -0.35 | -0.21 |
|  | Symptom would allow MOST of your daily activities | 0.25 | 0.20 | 0.08 | 0.08 | **0.29*** | **0.328** |
|  | Symptom would allow SOME of your daily activities | **0.51*** | 0.18 | -0.09 | 0.12 | -0.09 | **-0.37*** |
|  | Symptom would allow NONE of your daily activities | **0.72*** | 0.21 | 0.25 | **0.31*** | 0.15 | 0.26 |
| Duration of illness | Several days | -0.17 | -0.24 | -0.27 | 0.02 | -0.21 | -0.31 |
|  | Several weeks | 0.07 | -0.11 | -0.04 | 0.02 | -0.03 | -0.07 |
|  | Several months | 0.39 | 0.13 | 0.09 | 0.18 | 0.18 | 0.25 |
|  | Remainder of life | -0.30 | 0.22 | 0.22 | -0.23 | 0.07 | 0.13 |
| Vaccine effectiveness (VE) | 20% | **-1.10*** | **-0.95*** | **-0.71*** | - | - | - |
|  | 70% | **0.42*** | 0.22 | -0.12 | - | - | - |
|  | 95% | 0.46 | 0.06 | 0.24 | - | - | - |
|  | 99% | 0.23 | **0.66*** | 0.58 | - | - | - |
| Risk of illness without vaccination | 30 in 100,000 | -0.21 | -0.04 | -0.27 | - | - | - |
|  | 350 in 100,000 | -0.22 | 0.12 | 0.40 | - | - | - |
|  | 7,000 in 100,000 | 0.21 | 0.00 | 0.01 | - | - | - |
|  | 25,000 in 100,000 | 0.22 | -0.08 | -0.14 | - | - | - |
| Risk of death without vaccination | 0.06 in 100,000 | **-0.86*** | -0.13 | -0.05 | - | - | - |
|  | 250 in 100,000 | -0.05 | -0.13 | 0.03 | - | - | - |
|  | 1,260 in 100,000 | **0.57*** | 0.38 | 0.03 | - | - | - |
|  | 4,500 in 100,000 | 0.35 | -0.11 | 0.00 | - | - | - |
| Risk of severe side effects | 10 in 100,000 | -0.05 | 0.08 | -0.02 | -0.15 | 0.01 | 0.02 |
|  | 1,000 in 100,000 | 0.05 | -0.08 | 0.02 | 0.15 | -0.01 | -0.02 |
| Length of time vaccine available | 1 year | -0.36 | -0.14 | -0.24 | -0.13 | -0.05 | **-0.34*** |
|  | 5 years | 0.42 | -0.03 | 0.02 | **0.58*** | **0.38*** | **0.45*** |
|  | 15 years | 0.11 | **0.46*** | 0.25 | -0.15 | 0.27 | 0.05 |
|  | 30 years | -0.18 | -0.29 | -0.02 | -0.30 | **-0.60*** | -0.16 |
| Location | Doctor's office | 0.00 | -0.18 | -0.13 | 0.25 | 0.21 | 0.22 |
|  | Community or Public Health Clinic | 0.03 | 0.35 | 0.41 | **0.72*** | **0.81*** | **0.69*** |
|  | School | 0.23 | -0.02 | 0.06 | 0.10 | -0.10 | 0.06 |
|  | Clinic within a retail store / pharmacy | -0.27 | -0.14 | -0.34 | **-1.07*** | **-0.92*** | **-0.96*** |
| Time | 10 minutes | -0.13 | 0.19 | -0.04 | 0.03 | 0.11 | -0.10 |
|  | 20 minutes | 0.38 | -0.30 | -0.30 | -0.08 | **-0.32*** | -0.28 |
|  | 40 minutes | **-0.61*** | 0.37 | 0.45 | -0.16 | **0.43*** | **0.44*** |
|  | 2 hours | 0.36 | -0.25 | -0.11 | 0.21 | -0.22 | -0.07 |
| Healthcare provider type | Doctor | 0.25 | -0.23 | **-0.72*** | **-0.38*** | **-0.50*** | **-0.91*** |
|  | Nurse | 0.31 | -0.08 | -0.48 | -0.07 | **-0.37*** | **-0.44*** |
|  | Physician Assistant (PA) | -0.70 | 0.06 | 0.72 | -0.16 | 0.09 | 0.45 |
|  | Pharmacist | 0.15 | 0.24 | **0.49*** | **0.61*** | **0.78*** | **0.91*** |

Table S7 cont. Log odds for alternative model specifications

|  |  | Model 1:  Risk of Illness x VE  Risk of Death x VE | | | Model 2:  Risk of Illness x VE  Risk of Death x VE  Risk of illness and death, VE removed | | |
| --- | --- | --- | --- | --- | --- | --- | --- |
| Attribute | Level | Adults | Adolescents | Parents of adolescents | Adults | Adolescents | Parents of adolescents |
| PCP recommendation | PCP recommended NOT to receive vaccine | -0.08 | -0.16 | -0.56 | **-0.34*** | **-0.49*** | **-0.59*** |
|  | PCP never talked about the vaccine | **-1.32*** | -0.28 | -0.12 | **-0.50*** | -0.23 | -0.17 |
|  | PCP did not make specific recommendation | **0.86*** | -0.52 | -0.28 | **0.69*** | -0.23 | 0.10 |
|  | PCP said should think about getting vaccinated | 0.28 | 0.01 | -0.11 | -0.09 | 0.00 | -0.19 |
|  | PCP said it is very important to get vaccinated | 0.26 | **0.95*** | **1.07*** | 0.25 | **0.96*** | **0.85*** |
| Cost after insurance | $10 | 0.42 | -0.16 | -0.39 | 0.27 | -0.23 | -0.19 |
|  | $25 | **0.80*** | 0.01 | 0.07 | 0.39 | -0.31 | -0.02 |
|  | $50 | **0.75*** | 0.00 | -0.08 | -0.02 | -0.12 | -0.21 |
|  | $75 | 0.20 | -0.06 | **0.67*** | **0.54*** | **0.55*** | **1.09*** |
|  | $100 | 0.65 | 0.49 | -0.36 | 0.59 | 0.43 | -0.12 |
|  | $200 | **-0.71*** | -0.26 | 0.06 | -0.36 | -0.01 | 0.00 |
|  | $500 | -0.41 | 0.24 | -0.30 | **-0.72*** | **-0.42*** | **-0.71*** |
|  | $1,000 | **-1.71*** | -0.26 | 0.33 | **-0.69*** | 0.10 | 0.15 |
| Respondent gender | Male | 0.05 | -0.04 | 0.16 | 0.06 | -0.05 | 0.15 |
|  | Female | -0.05 | 0.04 | -0.16 | -0.06 | 0.05 | -0.15 |
| Median age category | Below median | -0.11 | 0.15 | 0.08 | -0.11 | 0.15 | 0.08 |
|  | At or greater than median age | 0.11 | -0.15 | -0.08 | 0.11 | -0.15 | -0.08 |
| Respondent education | < Bachelor’s degree | 0.13 | - | -0.08 | 0.14 | - | -0.11 |
|  | Bachelor’s degree and higher | -0.13 | - | 0.08 | -0.14 | - | 0.11 |
| Region | Northeast | -0.10 | -0.26 | -0.05 | -0.08 | -0.23 | -0.07 |
|  | Midwest | 0.05 | 0.30 | -0.01 | -0.07 | 0.30 | -0.04 |
|  | South | -0.04 | -0.07 | -0.21 | -0.04 | -0.04 | -0.24 |
|  | West | 0.08 | 0.03 | 0.27 | 0.20 | -0.03 | 0.34 |
| Race/ethnicity | White, non-Hispanic | -0.22 | -0.32 | -0.46 | -0.14 | -0.33 | -0.44 |
|  | Non-white, non-Hispanic | 0.22 | 0.32 | 0.46 | 0.14 | 0.33 | 0.44 |
| Household income | <$50,000/yr | -0.24 | 0.11 | 0.24 | -0.23 | 0.11 | 0.24 |
|  | >=$50,000/yr | 0.24 | -0.11 | -0.24 | 0.23 | -0.11 | -0.24 |
| Employed | Yes | -0.01 | - | 0.23 | 0.00 | - | 0.20 |
|  | No | 0.01 | - | -0.23 | 0.00 | - | -0.20 |
| Side effect experience | Yes | -0.44 | -0.27 | -0.32 | -0.37 | -0.27 | -0.30 |
|  | No | 0.44 | 0.27 | 0.32 | 0.37 | 0.27 | 0.30 |
| Risk of death X VE† | 0.06/100000 x 20% | 0.78 | 0.40 | 1.01 | 0.26 | 0.33 | 0.63 |
|  | 0.06/100000 x 70% | 0.86 | -0.42 | -0.39 | -0.05 | -0.53 | -0.07 |
|  | 0.06/100000 x 95% | -0.21 | -0.05 | -0.30 | 0.59 | 0.25 | -0.03 |
|  | 250/100000 x 20% | -0.88 | 0.20 | -0.02 | -0.42 | 0.14 | -0.12 |
|  | 250/100000 x 70% | -1.05 | 0.34 | 0.35 | -0.20 | 0.83 | 0.44 |
|  | 250/100000 x 95% | 0.69 | 0.34 | 0.36 | 0.31 | 0.18 | 0.60 |
|  | 1260/100000 x 20% | -1.78 | -0.28 | 0.35 | -0.55 | -0.05 | 0.44 |
|  | 1260/100000 x 70% | -0.18 | 0.63 | 0.52 | -0.19 | 0.22 | -0.16 |
|  | 1260/100000 x 95% | 1.64 | -0.31 | -0.89 | 0.29 | -0.69 | -0.78 |

Table S7 cont. Log odds for alternative model specifications

|  |  | Model 1:  Risk of Illness x VE  Risk of Death x VE | | | Model 2:  Risk of Illness x VE  Risk of Death x VE  Risk of illness and death, VE removed | | |
| --- | --- | --- | --- | --- | --- | --- | --- |
| Attribute | Level | Adults | Adolescents | Parents of adolescents | Adults | Adolescents | Parents of adolescents |
| Risk of illness X VE‡ | 30/100000 x 20% | -0.85 | 0.26 | 0.43 | 0.90 | 0.87 | 1.00 |
|  | 30/100000 x 70% | 0.27 | 0.26 | -0.99 | -0.21 | 0.06 | -1.05 |
|  | 30/100000 x 95% | -0.82 | -0.26 | 0.69 | -0.82 | -0.39 | 0.32 |
|  | 350/100000 x 20% | 0.58 | -0.64 | -1.02 | -0.41 | -1.13 | -0.97 |
|  | 350/100000 x 70% | -1.03 | -0.49 | 0.27 | -0.42 | -0.08 | 0.34 |
|  | 350/100000 x 95% | 0.82 | 1.01 | 1.13 | 1.11 | 1.20 | 0.99 |
|  | 7000/100000 x 20% | 0.37 | 0.18 | 0.16 | -0.11 | 0.07 | -0.08 |
|  | 7000/100000 x 70% | -0.04 | 0.11 | 0.42 | 0.15 | -0.12 | 0.52 |
|  | 7000/100000 x 95% | 0.26 | -0.62 | -1.21 | -0.12 | -0.60 | -0.92 |

*Indicates that the coefficient is significantly greater than zero (p<0.05). Positive levels that are significantly greater than zero indicate that respondents were more likely to choose a profile with that level relative to the mean, and negative levels that are significantly lower than zero indicate that respondents were less likely to choose a profile with that level relative to the mean.
